# Bayesian accrual prediction for interim review of clinical studies: open source R package and smartphone application

**DOI:** 10.1186/s13063-016-1457-3

**Published:** 2016-07-22

**Authors:** Yu Jiang, Peter Guarino, Shuangge Ma, Steve Simon, Matthew S. Mayo, Rama Raghavan, Byron J. Gajewski

**Affiliations:** Division of Epidemiology, Biostatistics, and Environmental Health, School of Public Health, University of Memphis, Memphis, TN 38152 USA; Cooperative Studies Program, VA Connecticut Healthcare System, West Haven, CT 06516 USA; Statistical Center for HIV/AIDS Research Prevention, Fred Hutchinson Cancer Research Center, Seattle, WA 98109 USA; Department of Biostatistics, School of Public Health, Yale University, New Haven, 06520 USA; P.Mean Consulting, Leawood, KS 66224 USA; Department of Biomedical and Health Informatics, University of Missouri at Kansas City, Kansas City, MO 64110 USA; Department of Biostatistics, University of Kansas Medical Center, Kansas City, KS 66160 USA; The University of Kansas Cancer Center, Kansas City, KS 66160 USA

**Keywords:** Bayesian methods, Smartphone application, Statistical software, Subject accrual

## Abstract

**Background:**

Subject recruitment for medical research is challenging. Slow patient accrual leads to increased costs and delays in treatment advances. Researchers need reliable tools to manage and predict the accrual rate. The previously developed Bayesian method integrates researchers’ experience on former trials and data from an ongoing study, providing a reliable prediction of accrual rate for clinical studies.

**Methods:**

In this paper, we present a user-friendly graphical user interface program developed in R. A closed-form solution for the total subjects that can be recruited within a fixed time is derived. We also present a built-in Android system using Java for web browsers and mobile devices.

**Results:**

Using the accrual software, we re-evaluated the Veteran Affairs Cooperative Studies Program 558— ROBOTICS study. The application of the software in monitoring and management of recruitment is illustrated for different stages of the trial.

**Conclusions:**

This developed accrual software provides a more convenient platform for estimation and prediction of the accrual process.

**Electronic supplementary material:**

The online version of this article (doi:10.1186/s13063-016-1457-3) contains supplementary material, which is available to authorized users.

## Background

Subject recruitment is critical and often very challenging in clinical research studies. Investigators frequently overestimate the pool of available subjects and underestimate the time needed to achieve the proposed sample size for their studies. This is known as Lasagna’s law [1] and as Muench’s third law [2]. Studies have shown that more than 80 % of clinical trial studies ran longer than their original accrual time goals [3]. A delay in study participant recruitment or an insufficient sample size can have serious deleterious consequences. Extending the recruitment time frame leads to increased costs, while a delay in study completion can lower the scientific impact or relevance. If the proposed sample size is not achieved, the study may be seriously underpowered. Therefore, it is important for researchers to monitor the accrual rate closely throughout the enrollment period of a study.

A number of studies have been published to model and predict patient accrual process. Both Barnard et al. [4] and Zhang and Long [5] recently reviewed prediction methods. The available accrual models include unconditional and conditional model, Poisson-based models, Brownian-motion-based models, and Bayesian models [4, 5]. Both Barnard et al. [4] and Zhang and Long [5] addressed the Bayesian methods developed by Gajewski et al. [6]. The Bayesian approach can utilize researchers’ previous experience from similar studies or clinical opinion and incorporate them as prior knowledge. When actual accrual data are available, the predictive distribution of the accrual data becomes the weighted average of the prior distribution and the actual observed data. As more data are collected, the weighting of the current observed data will increase while the weighting of prior information will decrease [6]. Such an approach provides an effective assessment of the accrual process.

Developing statistical methods are always important; however, providing reliable tools should also be a priority [7]. Typically, a clinical trial study team consists of clinicians, biostatisticians, project managers, software programmers, etc. Apart from biostatisticians, the majority of the other team members might not be familiar with the Bayesian framework, and it is challenging for them to use the algorithm for accrual prediction directly. Therefore, a simple and easy-to-use interface is in demand. One of the most commonly used and convenient approaches is to adopt the Bayesian computation into R, which is a free software environment for statistical analysis [8].

Although R and its packages are very popular in the area of statistics, many clinicians and project managers might not be familiar with them. A web calculator plus a smartphone application will make the tool more user-friendly and more convenient.

In this study, we translate our prediction model into an R *accrual* package. To make it easier, we applied the graphical user interface options in R, which only require researchers to input the original design information and the updated accrual data using simple point-and-click methods [8, 9]. The *accrual* package has been promoted on the RCRAN and is ready to be used by both statisticians and clinical researchers in evaluating and monitoring their subject accrual. We also derived a closed-form solution for the posterior prediction of the remaining subjects that can be recruited within a fixed time period, where the subject count is modeled as negative binomial. The credible interval for patient recruitment then can be calculated using a normal approximation for negative binomial. In a recent study [10], we also derived a closed-form solution for the posterior prediction of accrual with an inverse beta distribution. Based on the closed form, we developed a web browser and an Android version of the accrual calculator, which can be easily installed and used by a smartphone carrier.

The potential use of the accrual software for management and evaluation of the recruitment is discussed using the existing clinical studies conducted by the Department of Veterans Affairs Cooperative Studies Program Coordinating Center at West Haven, Connecticut [11, 12]. The application of the software is discussed in three aspects: (1) initial planning of the study, (2) interim review of the recruitment progress, and (3) evaluation of the site performance.

## Methods

### The general method and the closed form

In a clinical study, assume that the investigator needs to recruit *n* subjects. Based on previous trial experience and the potential available patient population, the investigator plans to finish recruitment in *T* days. Suppose that the trial starts at time *t*_0_, and that new patients enter the study sequentially, at *t*_1_, *t*_2_, *t*_*m*_ … Then the waiting time for each successive patient is calculated as

$$ {w}_i = {t}_i - {t_i}_{-1}, $$

and *w*_*i*_ is assumed to follow an exponential distribution, that is

$$ {w}_i\sim exp\left(\theta \right), $$

in which *θ* represents the average accrual time for the *i*th subject. To apply the Bayesian constant accrual model [6], we assume that the prior distribution of *θ* is inverse gamma, that is

$$ \theta \sim IG\left(nP,\kern0.28em TP\right), $$

where *P* is defined as the investigator’s confidence of finishing the trial in the original planned time, measured on a 0–1 scale [6]. In the process of a trial, suppose that *m* subjects have been collected within time *T*_*m*_(*T*_*m*_ = ∑_*i*= 1_^*m*^*w*_*i*_). Then the posterior distribution for *θ* is

1 $$ f\left(\theta \Big|m,{T}_m\right)=\frac{{\left(TP+{T}_m\right)}^{nP+m}\;}{\Gamma \left(nP+m\right)}{\theta}^{-\left(nP+m+1\right)}{e}^{-\frac{TP+{T}_m}{\theta }} $$

Using R, the Bayesian method for simple accrual [6] can easily be conducted using simulations. To speed up the calculation, it is better to develop a closed-form solution that can be used in Java. For fixed *T*, assuming that the rest of subjects can be recruited after time *T*_*m*_ are

$$ \eta \sim \mathrm{P}\mathrm{o}\mathrm{i}\left(\frac{T-{T}_m}{\theta}\right), $$

then the posterior predictive distribution of *η* is

2 $$ \begin{array}{c}\kern1em g\left(\eta \right)={\displaystyle {\int}_0^{\infty}\frac{{\left(TP+{T}_m\right)}^{nP+m}\kern0.28em }{\Gamma \left(nP+m\right)}{\theta}^{-\left(nP+m+1\right)}{\mathrm{e}}^{-\frac{TP+{T}_m}{\theta }}\frac{{\left(\frac{T-{T}_m}{\theta}\right)}^{\eta }}{\eta !}{\mathrm{e}}^{-\frac{T-{T}_m}{\theta }}\mathrm{d}\theta}\kern1em \\ {}\kern1em =\frac{{\left(TP+{T}_m\right)}^{nP+m}{\left(T-{T}_m\right)}^{\eta}\kern0.28em }{\Gamma \left(nP+m\right)\eta !}{\displaystyle {\int}_0^{\infty }{\theta}^{-\left(nP+m+\eta +1\right)}{\mathrm{e}}^{-\frac{TP+T}{\theta }}\mathrm{d}\theta}\kern1em \end{array} $$

3 $$ =\frac{{\left(TP+{T}_m\right)}^{nP+m}{\left(T-{T}_m\right)}^{\eta}\Gamma \left(nP+m+\eta \right)}{{\left(TP+T\right)}^{\mathrm{nP}+\mathrm{m}+\eta}\Gamma \left(nP+m\right)\eta !}. $$

Define

4 $$ p=\frac{TP+{T}_m}{TP+T} $$

and

$$ r=nP+m. $$

Then

$$ \left(\eta \right)={p}^r{\left(1-p\right)}^{\eta}\frac{\Gamma \left(\mathrm{r}+\eta \right)}{\Gamma (r)\eta !}. $$

This formula shows that the posterior predictive distribution of *η* is negative binomial:

NB(*r*,*p*).

As discussed by Jiang et al. [10], the closed form of the time frame of accrual shows an inverse beta distribution. We can use a normal approximation approach (Additional file 1), which can greatly accelerate the speed of calculation. The normal approximation works well if *r* is large and *p* is neither too small nor too large. To meet the requirement, we recommend that at the very beginning of the trial, when *m* and *T*_*m*_ are zero or small, the prior *P* should be relatively large (e.g., 0.5). After the trial starts, for example, when ${T}_m=\frac{T}{2}$, *p* will be in the range of 0.5 and 0.75. The value of prior *P* will almost have no effect on the normal approximation.

The derivation of the closed form also makes it possible to adopt the Bayesian constant accrual model in Java, which lacks built-in sampling algorithms.

### R accrual package, web-based calculator and smartphone applications

**Fig. 1:**
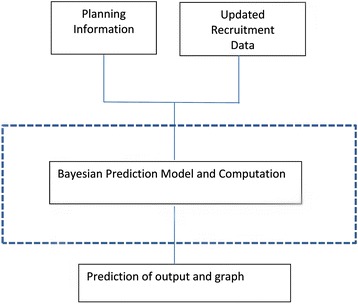
The flowchart of the accrual software

The objective of the package development is to adopt the Bayesian computation into R, which is ‘a free software environment for statistical computing and graphics’ [8]. As shown in Fig. 1, the entire Bayesian prediction model computation is in the background [6]. The design information includes the total time proposed for recruitment and the total sample size needed for the study, which are typically included in the protocol and required by institutional review boards. The developed software has three major functions: (a) to provide the estimate of the total number of subjects that the trial will have recruited within the planned time frame, (b) to provide the time frame that the trial will successfully recruit enough number of subjects, and (c) to produce diagnostic panel plots of the actual accrual data, such as the cumulative accrual plot and the distribution of the accrual etc.

**Fig. 2:**
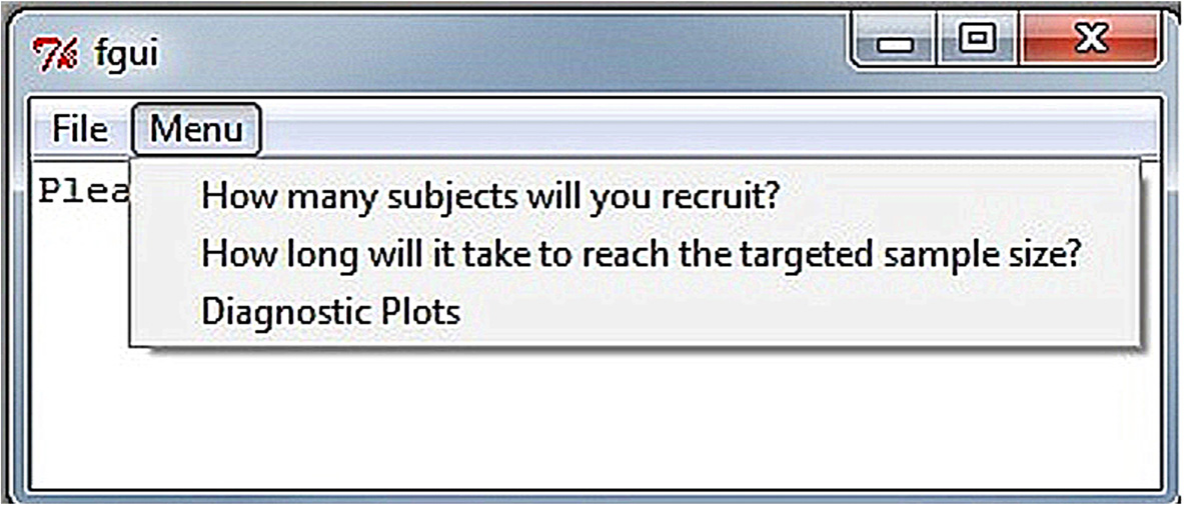
The main menu of the R *accrual* package with three options

The developed R *accrual 1.1* package has been promoted to R (> = 2.8.0) and is available for download from the Comprehensive R Archive Network. It includes an example data set, three major functions (described next), and a graphical user interface that provides a menu-driven access to these functions in R (Fig. 2). These three major R functions are *accrual.n*, *accrual.T* and *accrual.plots*. The function *accrual.n* calculates the prediction of the number of patients to be recruited in a fixed time. The function *accrual.T* predicts the time to reach targeted sample size. The function *accrual.plots* provides a panel of plots for data diagnostics. The *accrual.gui* provides an interface for the users to choose any of the three options as needed. The supplementary document provides a full menu on how to use the R package and how to interpret the results with examples.

As discussed here and in Jiang el al. [10], the closed form of the time frame of accrual is inverse beta, and the closed-form solution of the remaining subjects that can be recruited in a fixed time is distributed as negative binomial. Using a normal approximation for both of the above distributions can greatly accelerate the speed of calculation.

**Fig. 3:**
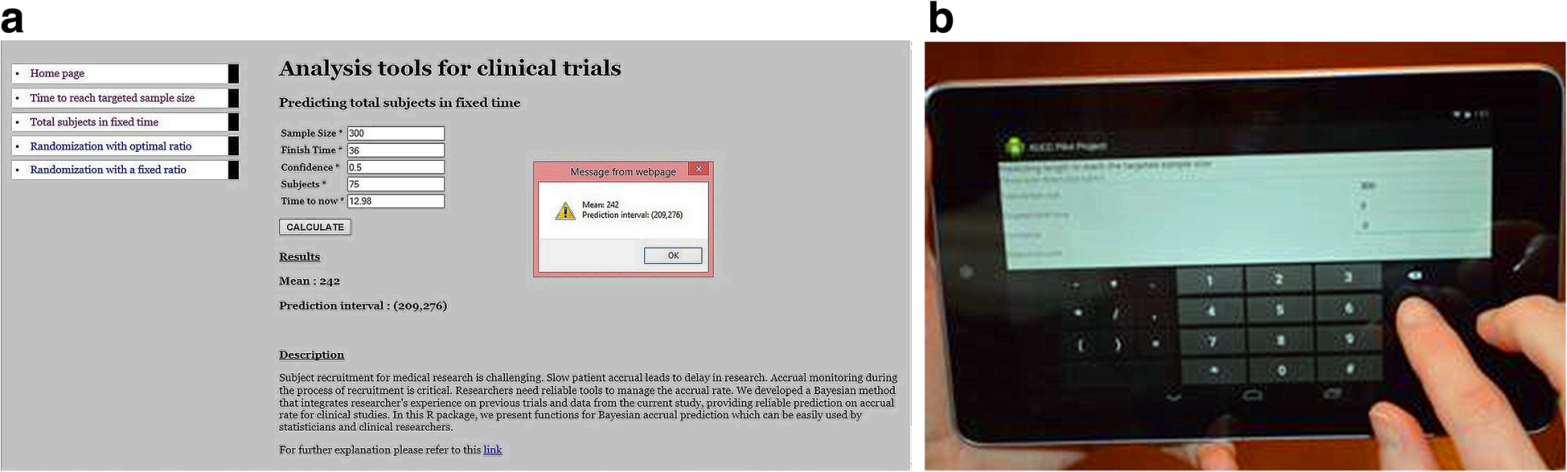
(**a**) An example of the use of accrual web-based software to calculate the number of patients can be recruited when one-quarter of the projected subjects has been recruited. (**b**) Use of the accrual smartphone application

Therefore, the normal approximation algorithm is adopted in Java and used for the accrual estimations. Our group developed a web-based accrual software using Java. An example of the use of this web calculator is shown in Fig. 3a. The link to the software can be found at http://biostat-pts.kumc.edu/velos/RPackages/Home.html.

With the development and widespread use of smartphones, the accrual smartphone application, compared with R packages or web-based application, can make the tool more user-friendly and convenient for clinical researchers. Using a closed-form solution for Bayesian accrual model and normal approximation, the methods are adopted into an Android application using Java. Figure 3b shows the use of an Android phone in the process of accrual monitoring.

## Results

Here we provide specific examples to illustrate how the accrual software can be used in the evaluation or management of a clinical trial. The ROBOTICS study was a randomized, multicenter, clinical trial to assess robot-assisted therapy for neuro-rehabilitation in chronic stroke patients [11]. The prediction of patient recruitment can be used at different stages of the study or for different purposes, such as study planning, interim review of the recruitment progress, and evaluation of the site performance.

### Initial planning of the study

**Fig. 4:**
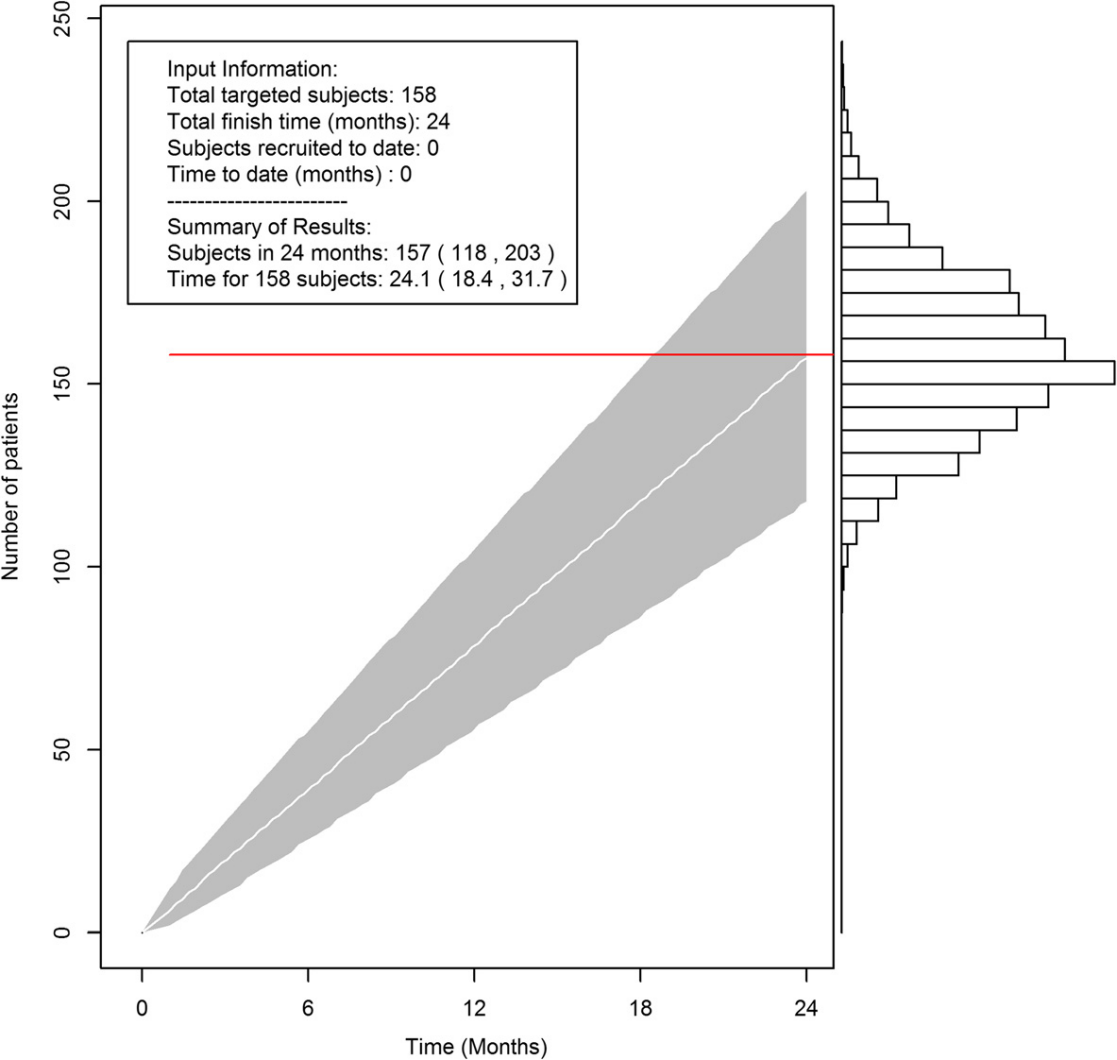
The R accrual package can be used at the beginning of the clinical trial to calculate the number of patients that can be recruited. The red line is the investigators’ original expected recruitment. The white line and gray tunnel are the projected accrual with 95 % credible interval

The target total sample size for ROBOTICS was 158, to achieve 90 % power of the protocol-defined effect size and variance assumptions estimated from the literature. Based on the investigators’ experience, the number of participating sites, and database estimates of the available study population, it was proposed to achieve the target sample size recruitment in 24 months. Using the accrual software, it is easy to project the recruitment with consideration for the randomness and uncertainty in the process. Figure 4 shows that the 95 % credible interval for patient accrual in 24 months is (118, 201). The time to recruit 158 patients could be between 18.5 months and 31.7 months, if one chooses *P* = 0.5. The graph and output can be used as a reference and guide for the investigator to plan the study, especially to estimate study budgets. As the total accrual in 24 months could be as low as 118, and the time to reach the full sample size could be as long as 31.7 months, the investigator would make some alternative plans, in case these extreme situations happens.

### Interim review of the recruitment progress

**Fig. 5:**
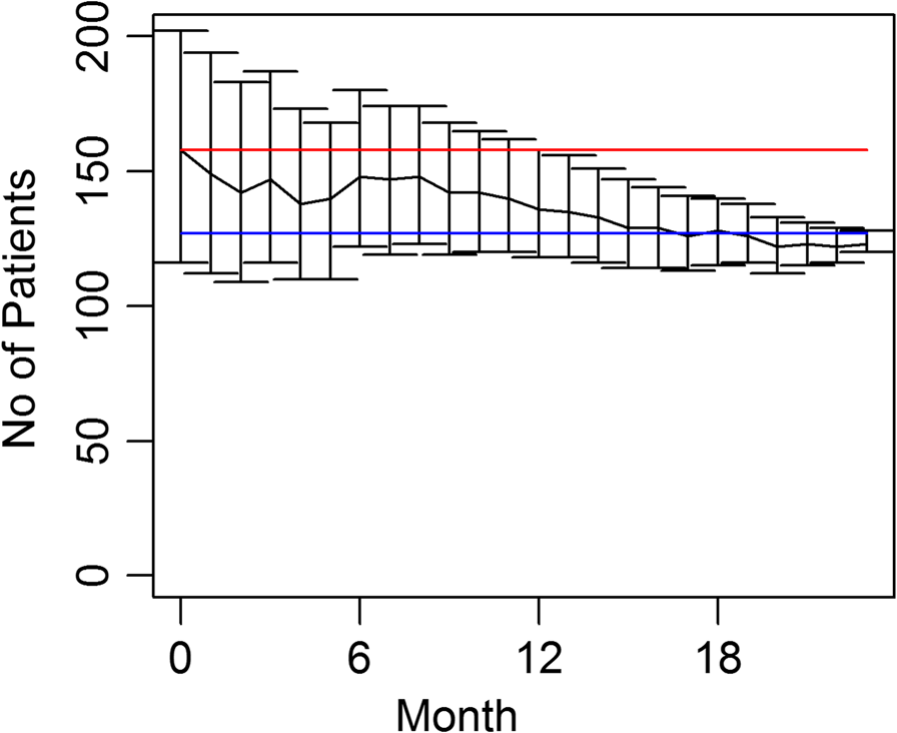
Projected patient recruitment with 95 % credible interval for the Robot study at each month: the red line indicates the proposed sample size *n* = 158 and the blue line is final recruitment *n* = 126

During the process of the clinical study, recruitment is almost always closely monitored. Usually, patient enrollments by participating site are summarized and reported on a weekly or monthly basis. The accrual software can be used for prediction at any time during the trial. The results will help investigators, sponsors, and oversight committees objectively evaluate the progress of the study. Figure 5 shows the projected accrual of patients at 24 months based on the current data using the software with *P* = 0.5. The figure clearly shows that, starting from months 13 it is highly probable that the study will not be able to recruit 158 subjects within the study time frame given the current recruitment process. The investigator should consider strategies to increase the accrual rate, such as adding one more study center, or changing the study protocols. The final recruitment ended up as 126 in 24 months, which was within the credible interval of all predictions shown in Fig. 5.

Overall, if the predicted accrual rate is so slow that it threatens the ability to achieve the proposed sample size and increases the trial duration of a study, this objective assessment will allow the study sponsor or data monitoring committees [3] to suggest mid-course corrections in the trial, such as adding additional centers to a multicenter study, hiring additional study coordinators to broaden the search for volunteers, or updating the inclusion or exclusion criteria. Conversely, the method can also prevent a researcher from overreacting to slow accrual at the beginning of the study. If the accrual is faster than planned, the prediction model can provide an estimated closure date to avoid unnecessary patient recruitment.

### Evaluation of the site performance

The accrual software is designed for single-site recruitment. However, most clinical studies are currently conducted at several sites. In the case of ROBOTICS, the study was conducted at four sites. The potential patient populations for each site and the experiences of site investigators are often different at each participating site. In the monitoring and management of recruitment in a multicenter trial, it is critical to evaluate the performance of each participating site separately. The sites that are significantly slow or inefficient in enrollment should have focused attention to try and improve the situation, and if no improvement is made during a ‘probation’ period, the prudent response can be to end that site’s participation in the study.

**Fig. 6:**
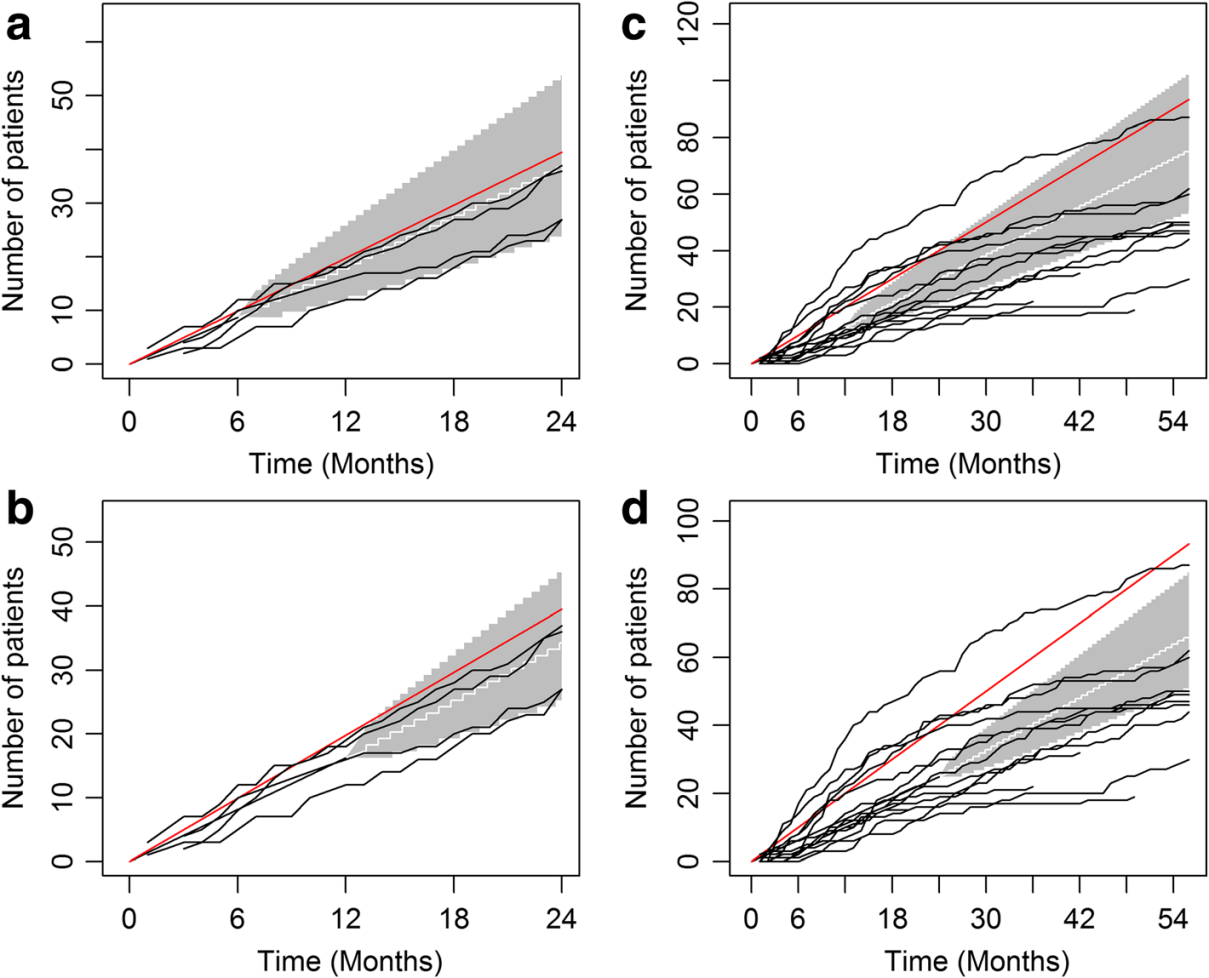
Evaluation of the patient recruitment for each of the four sites in the ROBOTICS study with enrollment data from (**a**) the first 6 months (**b**) the first 12 months, and the TEAM-AD study with enrollment data from (**c**) the first 12 months and (**d**) the first 24 months. The *red lines* are the investigators’ original expected recruitment for each site. The *white lines* and *gray* tunnels are the averages of recruitment for the four sites with projected accrual with 95 % credible interval. The *black lines* are the accumulated patient enrollment for each of the 14 sites

Figure 6a and 6b show the predicted average accrual for the ROBOTICS study when the trial started recruitment for 6 months and 12 months. The traditional and easiest method to evaluate the performance of an individual site is to use the red line, which is the investigators’ original expected recruitment, as a reference for each site by assuming that the accrual is constant without variations at each time points. As shown in Figure 6, only one site at the beginning of the trial is exceeding or even meeting the expectation, while the other three sites are below the target reference line throughout the trial. When compared with the projected recruitment target using accrual software, three of the sites fall within the 95 % credible interval, with only one site at or below the lower end of the confidence limits (the bottom line). As the Bayesian methods utilized the investigators’ original expectation and current recruitment data for all the sites, it is more reasonable in the evaluation of site performance compared with the traditional method.

The accrual software also applies to larger studies. Figure 6c and 6d display the recruitment process of another clinical trial study (TEAM-AD), a double-blind, placebo-controlled, randomized, clinical trial to assess the efficacy of α-tocopherol and memantine in Alzheimer’s disease [12]. The target sample size for the TEAM-AD was originally 840 patients, to be recruited from 14 sites over three years. At 12 months and 24 months, about 10 sites do not seem to meet the recruitment target when using traditional methods (red reference line). The results by Bayesian software show that the majority of sites perform in the 95 % confidence region, with approximately six sites well below expectation. Like the ROBOTICS study, the Bayesian method is more reasonable in evaluating site performance using both expected recruitment and current accrual data. Overall, the accrual software helps to recognize low performance. Early monitoring and identification of low performing sites will help study oversight committees make well informed decisions and work out efficient strategies to improve the enrollment of each site, which in turn improve the management of recruitment for the entire study.

## Discussion

Development of software can be as important as the development of novel statistical methods [7]. In this paper, we evaluated the completed ROBOTICS and the TEAM-AD clinical trials using the proposed software by demonstrating that the recruitment tool can be used at all stages of the study.

The R *accrual* packages, both the web-based and the smartphone application for patient accrual, are based on the assumption that the accrual rate is constant. Based on our previous findings [13], the assumption for constant accrual is reasonable and generally holds, especially for small single-site clinical trials conducted in an academic research institute. The performance of the constant accrual model has also been evaluated via real clinical studies and extensive simulation studies [10]. The general finding is that when the accrual is on target, a strong prior (a relative larger *P*, such as *P* = 0.5) performs better. When the accrual is off target, a weak prior (a smaller *P*, such as *P* = 0.01) works better in prediction. The constant accrual model performs well when the accrual rates vary slightly, for example, by being more slow at the beginning of a trial. Even in some situations where the model does not predict the completion time correctly, it can still recognize early that an accrual is off target.

In the constant accrual model, specification of the prior, *P* is critical. In general, if the investigators have rich experience in the study and follow the definition of *P* correctly to choose a reasonable value for *P*, it will be beneficial to model prediction. As illustrated in the simulation study, when the accrual is on target and the investigators have higher confidence, they tend to choose a larger *P,* which will lead to better prediction. When the accrual is slow, the investigators lack confidence and are more likely to choose a smaller *P*, which will also lead to a better prediction. In some situations where the users have a hard time specifying *P*, we suggest *P* =0.1 as a reasonable value to start with.

Overall, the software is robust when the assumption is only slightly violated [10, 13]. However, it should still be used with caution and it is worth examining the data distribution to check whether the assumptions are violated or not. In the current software, we only have diagnostic plots for data distribution. In the future, we will add more model diagnostic methods, such as testing the assumption for independence and sensitivity analysis [13]. We assume that the waiting time is distributed exponentially. A more general approach in the future is to model the waiting time using a Weibull distribution. For obvious non-constant accrual studies, we are working on linear piecewise regression models, where the accrual is divided into a certain number of stages. It has been shown that it is critical to choose the prior, *P*, in the prediction of accrual. To avoid poor choices of *P*, we have introduced adaptive priors, an accelerated prior and a hedging prior, which are shown to be more robust than a subjective prior when the constant accrual assumption is violated. The accrual software with these new priors will be available soon. In addition, the current accrual software is only designed for single-site clinical studies or the review of a single site. An upgrade version of the accrual software, which includes prediction for multisite recruitment, is under development.

## Conclusions

In summary, we present an R *accrual* package, and a built-in Android system using Java for web browser and mobile devices based on a previously developed Bayesian constant accrual model. The accrual software provides a convenient platform for researchers in the evaluation of the accrual process in clinical studies. We use specific examples to illustrate how the software can be used, as well as its strength and limitations. Future planned work will involve continued assessment of our assumptions. We also plan to build new models for both single-site and multisite clinical trials and to translate these methods into easy-to-use software for monitoring clinical trials.

## Abbreviations

ROBOTICS, robot-assisted upper limb neuro-rehabilitation; TEAM-AD, the trial of vitamin E and memantine in Alzheimer’s disease; Virginia, veterans’ affairs

## Additional file

Additional file 1: Illustration of use of R accrual package. (DOCX 917 kb)
