# Supplementary material for: Bayesian accrual prediction for interim review of clinical studies: open source R package and smartphone application
Source: Trials. 2016 Jul 22;17:336. doi: 10.1186/s13063-016-1457-3 (PMC4957321; doi:10.1186/s13063-016-1457-3)
Supplement: Additional file 1: — Illustration of use of R accrual package. (DOCX 917 kb) [file 13063_2016_1457_MOESM1_ESM.docx]

**Illustration of using R accrual package**

Suppose in a clinical trial, the researcher's original proposal is to recruit 158 patients in 2 years (24 months). Assuming that the investigator is 50% confident that the accrual can be done within the planned time, that is P=0.5, and that the trial has not been started, the estimation of total patients will be recruited in three years can be done using the function *accrual.n*,  *accrual.n (n=158,T=24,Tp=24, P=0.5,m=0,tm=0).*In addition, users can also choose to use the GUI window. Figure S2A shows the interface window for the users to estimate “How many patients will you recruit?”, which functions exactly as *accrual.n*. In this case, the “Targeted sample size” is 158. “Targeted finish time in months” is 24. The researcher can choose any confidence level (0 to 1) by using a slider or directly entering the confidence level in the blank directly. In the current case, the trial has not been started, therefore both “Subject recruited” and “Total months after started” are 0. Through either of the two approaches, the prediction of the accrual is shown in Figure S2B. The white line is the estimate of the prediction, with the grey tunnel as the prediction intervals. The histogram of estimated total accrual in 24 months is shown on the right. The horizontal line indicates the target sample size. On the top left corner of the figure, there are input information and the summary of the results. In this example, there will be 158 subjects recruited in 24 months, with 95% prediction interval (118, 203). It will take 24.1, with 95% prediction interval (18.4, 31.7) months for the investigators to recruit 158 subjects. The plots and the summary results will help the investigators and the IRBs to monitor and predict the progress of the clinical trial. Please be aware there may be a slight difference in the results between the displayed results and what will be obtained by the reader using the package. This is due to the using of simulation approach in the calculation. The trivial difference will not affect the interpretation of the results and the evaluation of the trial progress.

Using the R accrual package, the recruitment of the trial can be monitored all along the process. For example, if the trial is in progress with 40 subjects recruited in 8 months, the prediction can be done as either use *accrual.n* function or the interactive R window for data input, as shown in Figure S3A. Figure S3B shows the corresponding accrual plot and summary of the results. If 1/4 of total sample size, which is 40 subjects, recruited in 8 months, then the predicted total subjects can be recruited in 2 years is 135 with 95% prediction interval (111, 162).

In addition to estimating the total number of subjects in a fixed time, the investigators may also be interested in estimating the time frame to finish recruiting a certain number of subjects. It can be done by using function *accrual.T*, or the second option (Figure 1) “How long will it take to reach the targeted sample size?”. Similarly as the previous example, the researcher's original proposal is to recruit 158 patients in 24 months, and the researcher's confidence level is also 0.5. The R window interface and output are shown in Figure S4A and Figure S4B respectively. In Figure S4B, the summary of input information and output are shown on the left bottom corner. The vertical line shows the targeted time (24 months). As shown clearly in Figure 4B, the predicted time to finish recruiting 158 subjects is 27.8 months, with 95% predicted interval (23.4, 33.6). The predicted accrual is much slower than planned 24 months. The results indicated that both the IRBs and the investigator should pay special attention to the accrual rate as discussed above.

The Bayesian accrual model is based on the assumption that the distribution of waiting time is exponential and the rate of the accrual is constant. Violations of the assumption may lead to biased estimation. Therefore, it is useful to check whether data (w) meet the assumption and is suitable for the current method via option “Diagnostic Panel”. Figure S5 A shows the windows interface of this option, in which the researchers can load the raw time gap data through point and click. Figure S5B shows four figures that help to understand the data distribution. The figure on the top left is the exponential quantile plot, which checks whether the distribution of waiting times is exponential. The current plot shows that data are off from the straight line. The top right figure shows the histogram of the waiting times, where the red line is the theoretical exponential distribution. The figure of waiting time verse cumulative accrual time is shown on the bottom left, and the figure of total accrual verse cumulative accrual time is shown on the bottom right. Both of the graphs show that this trial is piecewise constant with slower accrual in the beginning and at the end.

**Figure S1** **The distribution NB (225, 0.5737037) (dotted line) and its corresponding Normal distribution approximations (solid line)**


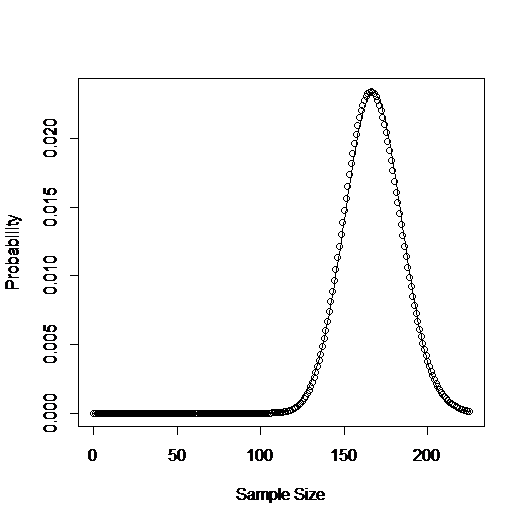


**Figure S2 An example of using R *accrual* package to calculate the number of patients can be recruited in the beginning of clinical trial. (A) The interactive R window for data input (B) The R output for summarized results and accrual plot.**

**(A)**


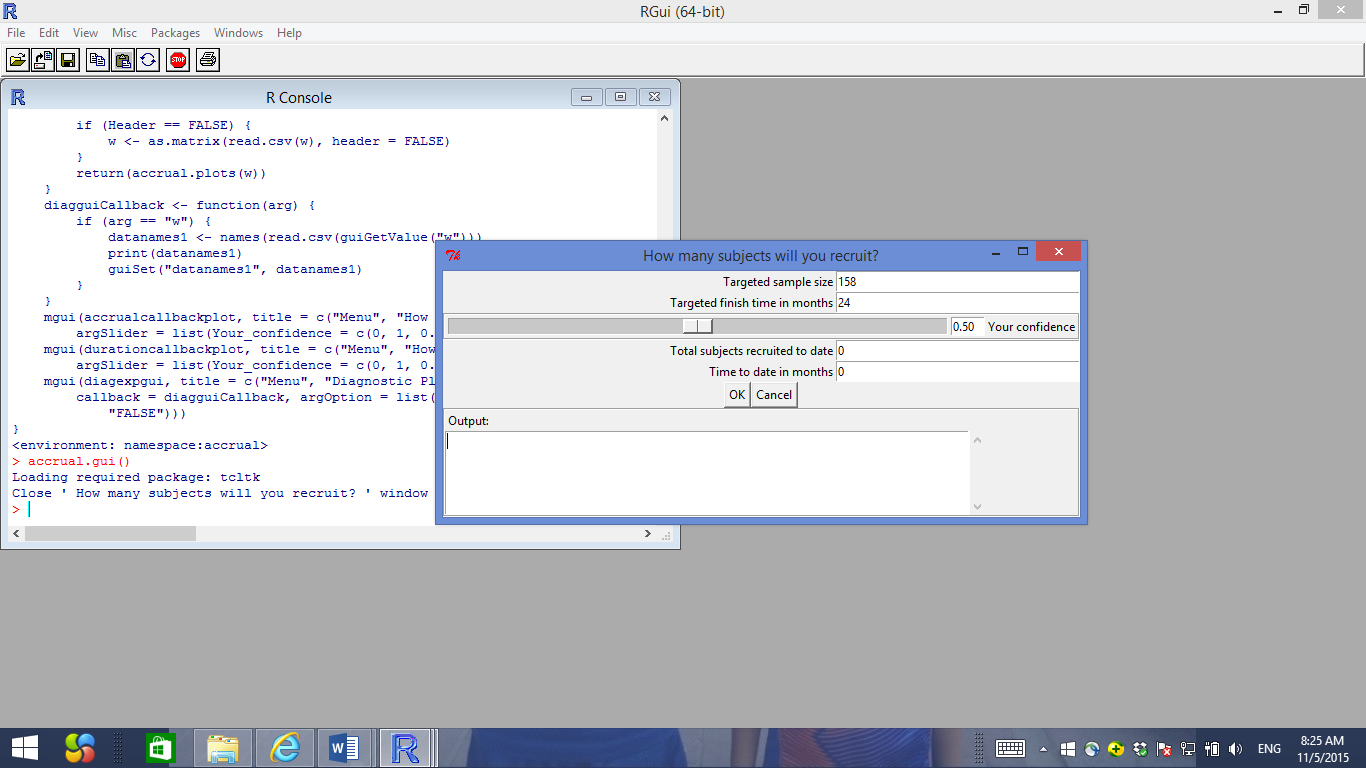


**(B)**


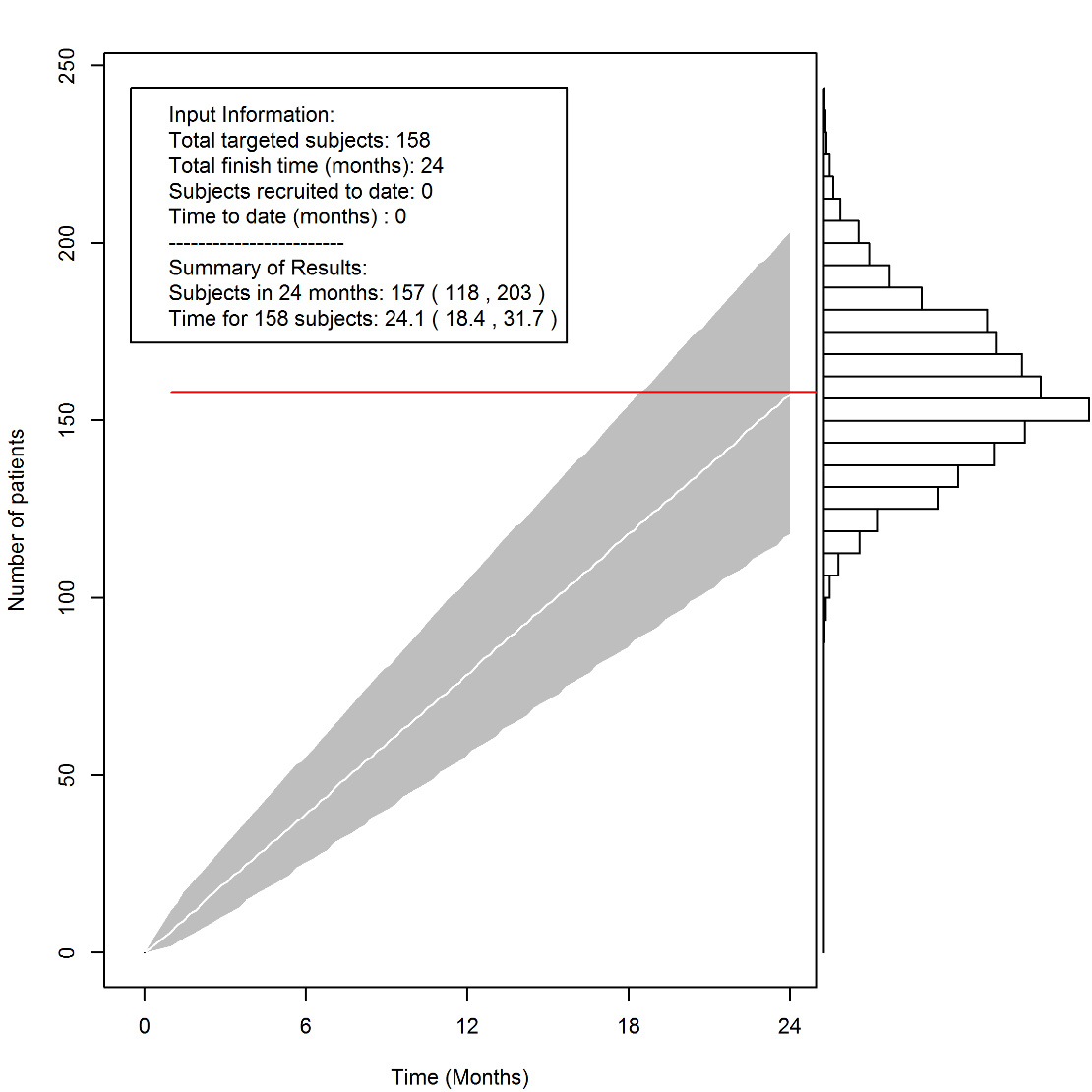


**Figure S3. An example of using R *accrual* package to calculate the number of patients can be recruited when 75 subjects has been recruited. (A) The interactive R window for data input (B) The R output for summarized results and accrual plot.**

**(A)**


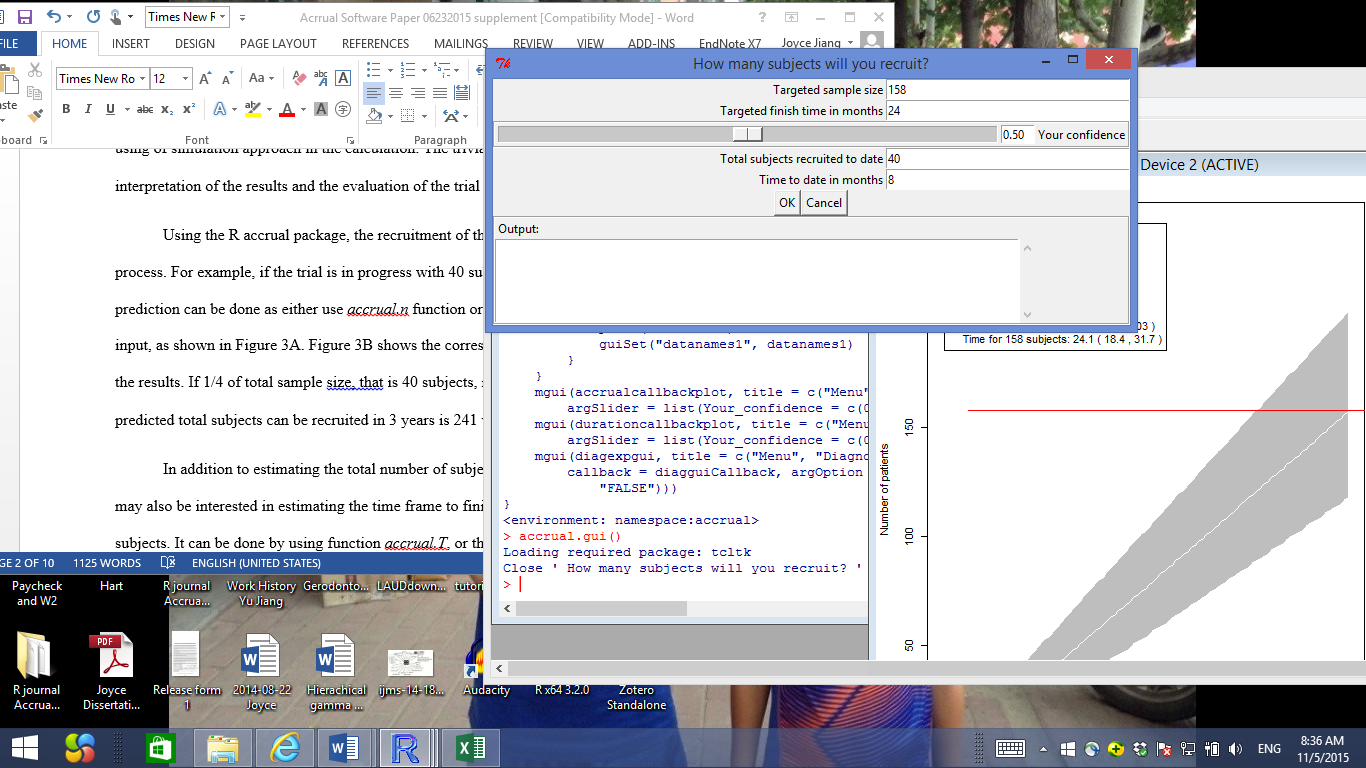


**(B)**


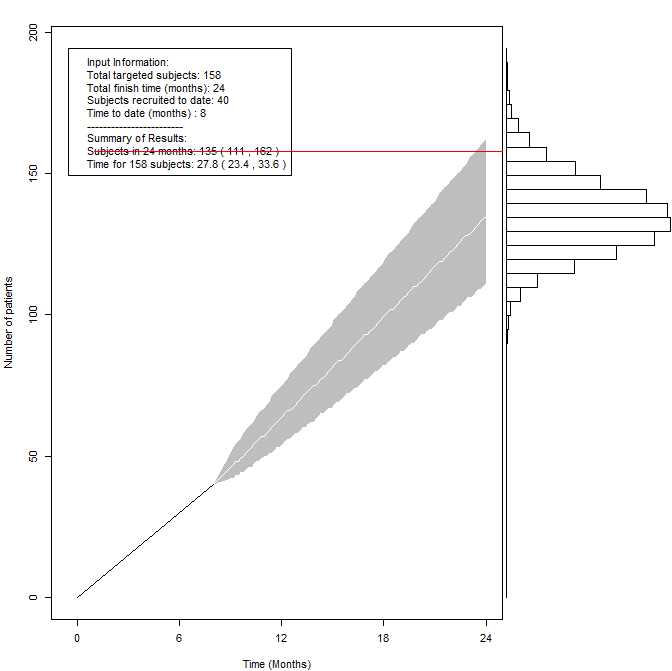


**Figure S4 An example of using R *accrual* package to calculate the time frame to reach the targeted sample size when 75 subjects has been recruited. (A) The interactive R window for data input (B) The R output for summarized results and accrual plot.**

**(A)**


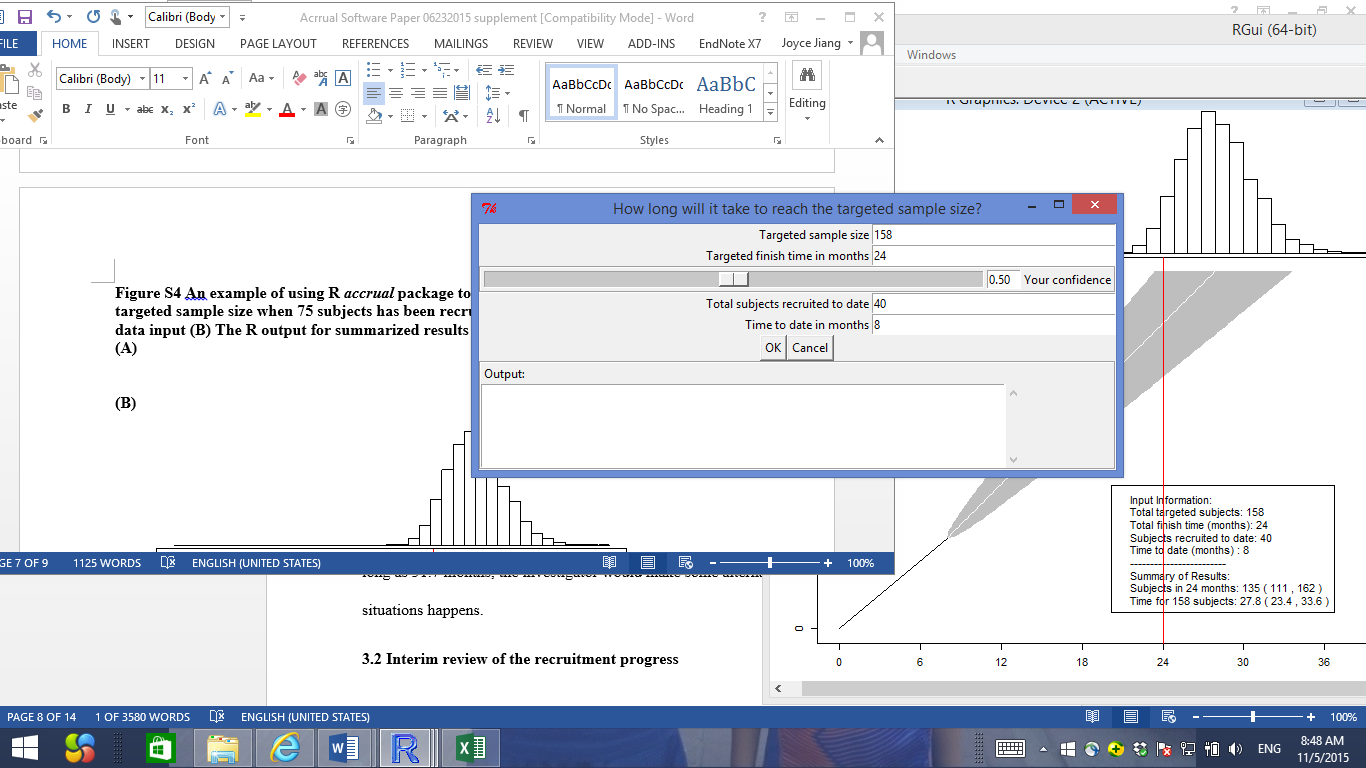


**(B)**

**Figure S5 An example of using R *accrual* package to check the distribution of accrual data (A) The interactive R window to input data (B) The R output for exponential quartile plot for waiting times (top left), the histogram of the individual waiting times (top right), waiting times verse cumulative accrual time (bottom left), and the number of subjects verse cumulative accrual time (bottom right).**

**(A)**


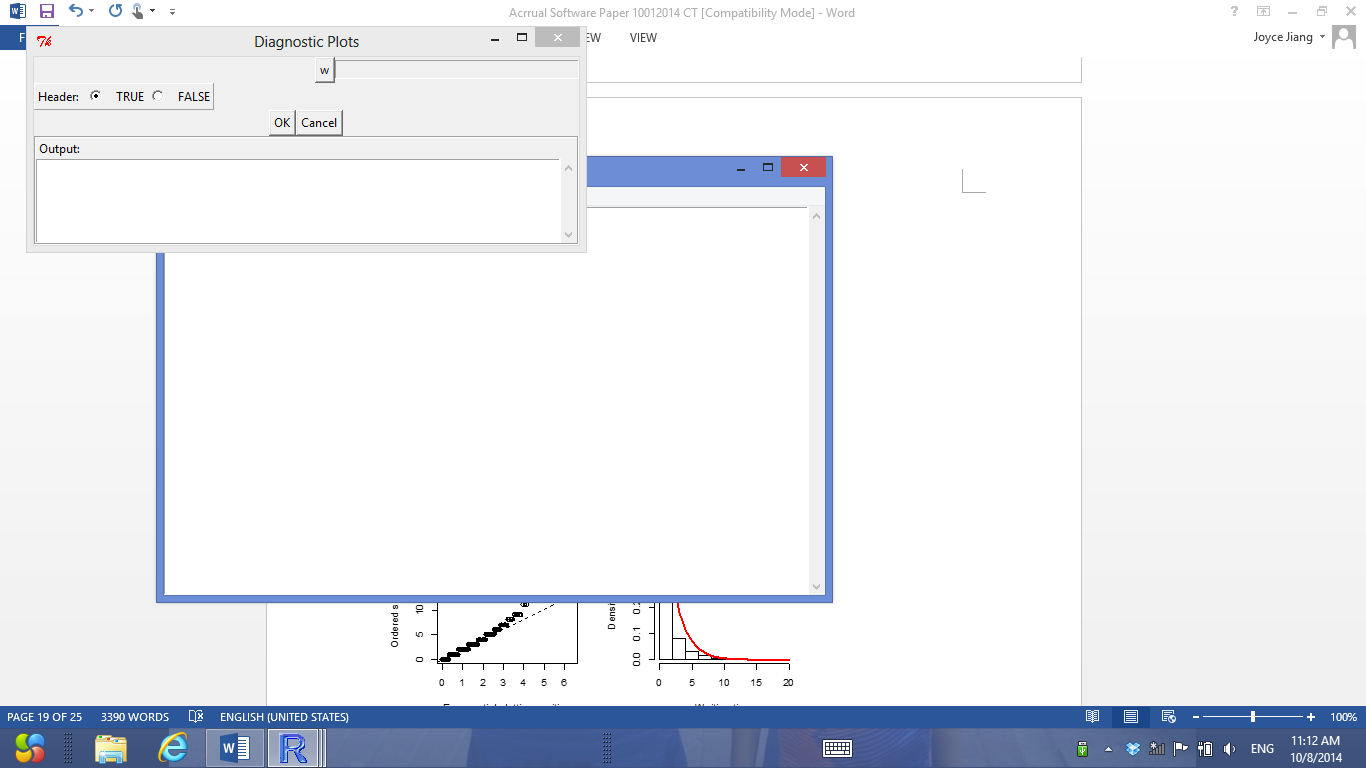


**(B)**
